# Acidosis Sensing Receptor GPR65 Correlates with Anti-Apoptotic Bcl-2 Family Member Expression in CLL Cells: Potential Implications for the CLL Microenvironment

**DOI:** 10.4172/2329-6917.1000160

**Published:** 2014-09-20

**Authors:** Ashley E Rosko, Karen S McColl, Fei Zhong, Christopher B Ryder, Ming-Jin Chang, Abdus Sattar, Paolo F Caimi, Brian T Hill, Sayer Al-Harbi, Alexandru Almasan, Clark W Distelhorst

**Affiliations:** 1 Division of Hematology-Oncology, University Hospitals Case Medical Center, Cleveland, Ohio, USA; 2 Division of Hematology-Oncology, Case Western Reserve School of Medicine, Cleveland, USA; 3 Department of Epidemiology and Biostatistics, Case Western Reserve University, Cleveland, OH, USA; 4 Department of Hematologic Oncology and Blood Disorders, Cleveland Clinic, Cleveland, Ohio, USA; 5 Department of Cancer Biology, Lerner Research Institute, Cleveland Clinic, Cleveland, Ohio, USA; 6 Case Comprehensive Cancer Center, Case Western Reserve University School of Medicine, Ohio, USA; 7 Division of Hematology, Department of Medicine, Ohio State University College of Medicine, Columbus, Ohio, USA

**Keywords:** Chronic lymphocytic leukemia, TDAG8T cell death associated gene, GPR65G protein-coupled receptor 65 Bcl-2B-cell Lymphoma 2, Microenvironment

## Abstract

The tumor microenvironment is generally an acidic environment, yet the effect of extracellular acidosis on chronic lymphocytic leukemia (CLL) is not well established. Here we are the first to report that the extracellular acid sensing G-protein coupled receptor, GPR65, is expressed in primary CLL cells where its level correlate strongly with anti-apoptotic Bcl-2 family member levels. GPR65 expression is found normally within the lymphoid lineage and has not been previously reported in CLL. We demonstrate a wide range of GPR65 mRNA expression among CLL 87 patient samples. The correlation between GPR65 mRNA levels and Bcl-2 mRNA levels is particularly strong (r=0.8063, p= <0.001). The correlation extends to other anti-apoptotic Bcl-2 family members, Mcl-1 (r=0.4847, p=0.0010) and Bcl-xl (r=0.3411, p=0.0252), although at lower levels of significance. No correlation is detected between GPR65 and levels of the pro-apoptotic proteins BIM, PUMA or NOXA. GPR65 expression also correlates with the favorable prognostic marker of 13q deletion. The present findings suggest the acid sensing receptor GPR65 may be of significance to allow CLL tolerance of extracellular acidosis. The correlation of GPR65 with Bcl-2 suggests a novel cytoprotective mechanism that enables CLL cell adaptation to acidic extracellular conditions. These findings suggest the potential value of targeting GPR65 therapeutically.

## Introduction

Among the adaptive hallmarks of cancer cells is the ability to survive and proliferate within a caustic tumor microenvironment [1]. Metabolic derangements that contribute to a nutrient poor, hypoxic and acidic milieu are well described in malignant proliferating tissue. Nearly 80 years ago, Warburg first described the metabolic shift from mitochondrial oxidative phosphorylation to glycolysis, a characteristic metabolic modification in cancer cells [2,3]. The upregulation of glycolysis, generating lactic acid paired with an increase in proton efflux, results in an abundance of acidic waste products within the extracellular space [4–6]. The culmination of this metabolic stress along with decreased clearance of metabolic waste products lends itself to an acidic extracellular environment [7,8]. Acidic conditions are variously implicated as contributing to drug resistance, DNA damage and repair, protein alterations, angiogenesis, and metastasis [9–11]. Acidosis has been detected in the tumor microenvironment by various methods utilizing microelectrodes or non-invasive imaging approaches, documenting a spectrum of acidic conditions that vary with each malignancy [12–14].

Cancer cells can respond to extracellular acidosis through a family of acid sensing G protein-coupled receptors, the natural ligand of which is the proton [15,16]. Members of this family include OGR1/ GPR68, GPR4, G2A, and GPR65/TDAG8. Upon proton binding to histidine residues in their extracellular exposed domain, these proteins signal downstream via G proteins [17,18]. GPR65 (G protein-coupled receptor 65), also known as TDAG8 (T cell death associated gene 8), is mainly expressed in the lymphoid lineage, although its expression has also been detected in certain non-lymphoid malignancies, including those of the kidney, ovary, colon and breast [19]. The oncogenic activity of GPR65 was recently demonstrated by Ihara et al., who found that its overexpression increases survival of cancer cells in a xenograft tumor model [20]. GPR65-induced cell growth and proliferation were abrogated by mutation of the proton-binding histidine residues in its extracellular domain. How GPR65 activation by extracellular acidosis promotes cancer cell survival has not been well defined.

We recently discovered that GPR65 signaling in response to extracellular acidosis increases survival of leukemia and lymphoma cell lines by up-regulating the anti-apoptotic proteins Bcl-2 and Bcl-xL [21]. Our studies placed cells under metabolic stress by depriving them of glutamine and/or glucose to mimic the adverse conditions typical of the microenvironment. Moreover, we found that GPR65 activates signaling via the MEK/ERK pathway to up-regulate these proteins and protect against cell death. Furthermore, as a potential translational correlative, extracellular acidosis induced Bcl-2/Bcl-xl level elevation, sensitizing lymphoma/leukemia cell lines to killing by the BH3-mimetic, ABT-737 [21]. This result suggests that extracellular acidosis promotes Bcl-2-dependence.

Here we are the first to report that GPR65 is expressed in primary human Chronic Lymphocytic Leukemia (CLL) cells and that GPR65 expression levels in these cells correlate strongly with expression levels of the anti-apoptotic Bcl-2 family members Bcl-2, Mcl-1, and Bcl-xl. Evidence is also presented indicating that extracellular acidosis may contribute to the elevated Bcl-2 levels typical of CLL cells. These findings are of interest in view of abundant evidence of the importance of the tumor microenvironment in the pathogenesis of CLL [22–24]. Current understanding of the CLL tumor microenvironment, primarily in lymph nodes and bone marrow, is focused mainly on interactions of CLL cells with T-lymphocytes, endothelial cells, nurse like cells, soluble elements, and stromal factors [25,26]. However, very little is known about the metabolic stresses and modulations in pH that CLL cells encounter within their microenvironment. The present findings suggest that GPR65 signaling in response to extracellular acidosis may augment the survival of CLL cells in the microenvironment. Our data provide a strong rationale for recognizing GPR65 as a viable therapeutic target for the treatment of CLL.

## Materials and Methods

### Cell isolation and culture

Peripheral blood samples from 87 patients with CLL were obtained in the outpatient clinic with the patients’ informed consent according to protocols approved by the University Hospital Case Medical Center Institutional Review Board and the Cleveland Clinic Institutional Review Board according to the Declaration of Helsinki. University Hospitals Case Medical Center study data were collected and managed using REDCap electronic data capture tools [27]. Lymphocytes were purified by Ficoll-Paque gradient centrifugation, washed in PBS, followed by suspension in ACK (ammonium-chloride-potassium) lysis buffer (Lonza). Samples were lysed for RNA or protein analysis. All primary CLL cells used for mRNA and protein analysis were freshly processed. CLL cells used for in vitro experiments were purified and then cultured in pH buffered DMEM –HEM (HEPES, EPPS, MES) and samples were immediately incubated after isolation where the pH was adjusted to 6.5 (low pH) or 7.5 (control pH), as recently described [21]. Cell viability was quantified based on exclusion of Trypan blue.

### Quantitative RT-PCR

Total RNA was isolated using Trizol/RNEasy reagent (Invitrogen, Carlsbad, CA) according to the manufacturer’s protocol. Isolated RNA was precipitated in isopropanol, washed in ethanol, and dissolved in RNase-free water. RNA concentration was determined by measuring absorbance at 260 and 280 nm. Total RNA was reverse transcribed using the TaqMan Gold RT-PCR kit (Applied Biosystems, Foster City, CA). Amplification was performed either utilizing the Universal TaqMan master mix and primer/probe sets specific for the genes of interest (Applied Biosystems) and examined on a Lightcycler 480 II (Roche), or alternatively amplified using the SYBR Green Master Mix (Applied Biosystems, N8080234 and 4309155) and examined on a 7500 Real-Time PCR system (Applied Biosystems), as previously described [28]. A purchased human B cell (CD19+) total RNA was used to establish a baseline for comparison between mRNA levels in CLL and a healthy subset (Miltenyi). Quantitative, real-time RT-PCR was performed by the relative quantification (2−ΔΔCt) method using β-actin as the reference mRNA.

### Protein analysis

Western Blotting was performed using standard laboratory methods as previously described [21]. Protein quantification was performed using Image J analysis.

### Statistical analysis

Distributions of GPR65 mRNA expression, apoptotic family members, and other quantitative variables were analyzed using box plots, Q-Q plots, and Shapiro-Wilk tests for normality. Logarithmic transformations were performed on highly skewed variables. The Pearson product-moment correlation coefficient was used in studying the association between GPR65 and Bcl-2 family members. The Pearson correlation coefficient was also used in determining the relationship between GPR65 and hematologic parameters. Simple linear regression analyses were used in studying the association between GPR65 expression and patient clinical characteristics such as age, sex, and Rai stage. The two-sample t-test was used in comparing Bcl-2 expression ratio and the GPR65 expression ratio. All statistical analyses were performed using STATA 11.0 software.

## Results

### Clinical data

Peripheral blood samples were collected for analysis from 87 individual CLL patients. Clinical and biologic data were analyzed for 76 of these patients (Table 1). Patients were mostly early Rai Stage (76% Stage 0–2), CD38 negative (58%), and Zap-70 negative (58%). More than half (57%) were untreated and managed with observation alone. Baseline laboratory data were gathered at the time of sample collection. Cytogenetic abnormalities included trisomy 12 in 20% of patients and deletion of the long arm of chromosome 13 in 53% of patients.

### GPR65 expression in primary CLL cells

**Figure 1:**
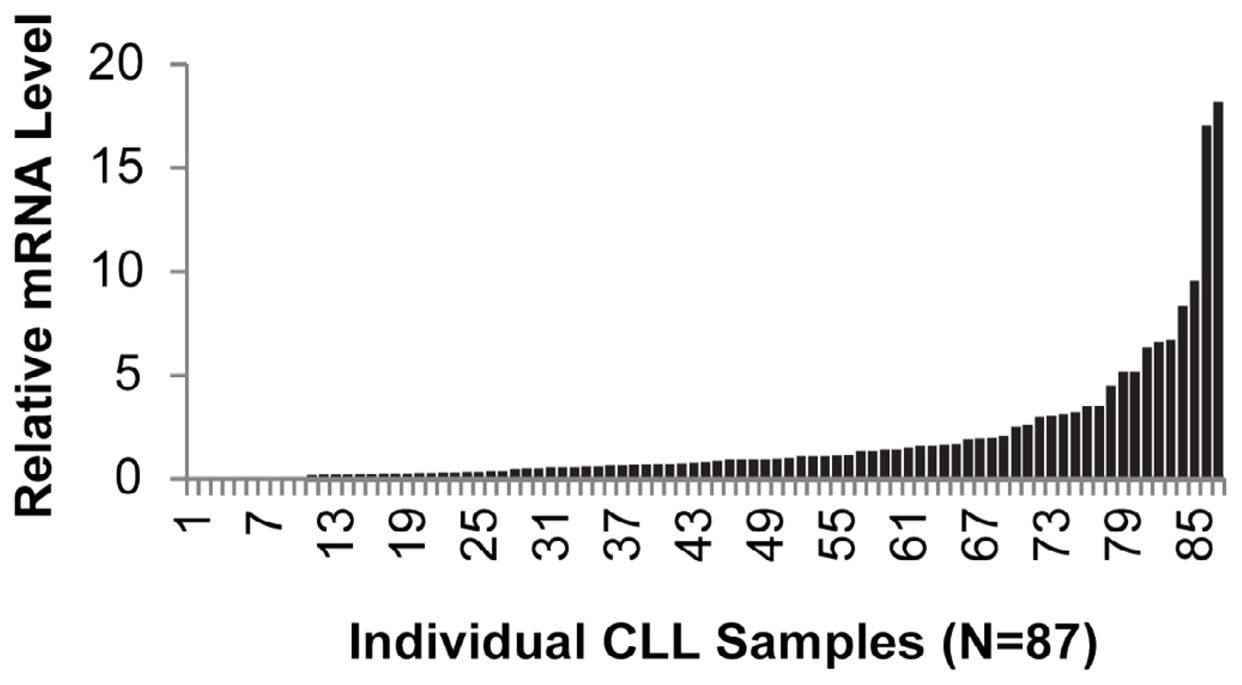
GPR65 mRNA expression profile in primary CLL cells. Basal GPR65 mRNA levels were measured by qRT-PCR in primary CLL cells (N=87 individual patients) relative to CD19+ healthy control cells. Levels range from 0.02 to 18.07 with a median expression of 0.71.

GPR65 mRNA expression levels were measured in primary CLL cells by qRT-PCR and normalized to corresponding levels in healthy human CD19+ B cell controls (Figure 1). Healthy CD19+ B-cell control clinical parameters include half male and half female, age distribution of 18–68 years, with a cell purity of >90%. GPR65 mRNA levels in isolated primary CLL samples range widely from 0.02 to 18.07 with a median mRNA expression of 0.71, relative to healthy human B cell controls. GPR65 levels were standardized using the respective expression of beta-actin. To investigate the potential relationship of known CLL prognostic factors to GPR65 expression, linear regression analysis was employed on all 87 samples, demonstrating that GPR65 expression correlates with 13q deletion, a favorable prognostic marker (Table 2). None of the other known CLL prognostic factors correlated with GPR65 expression.

### GPR65 levels correlate with levels of anti-apoptotic Bcl-2 family members in CLL cells

**Figure 2:**
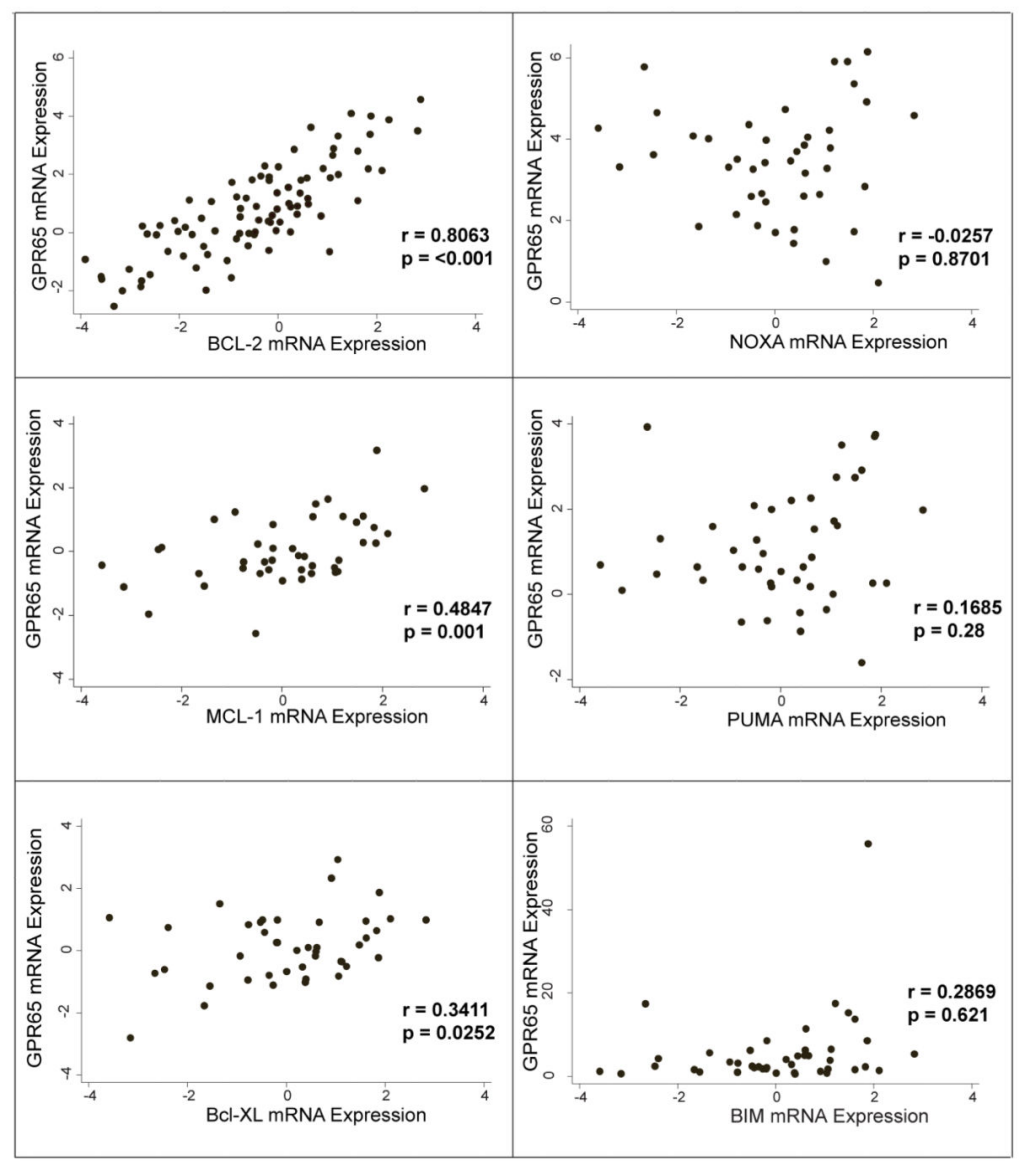
GPR65 correlates strongly with anti-apoptotic family members Bcl-2, Mcl-1 and Bcl-xl. Basal GPR65 mRNA levels measured by qRT-PCR in primary CLL cells were plotted versus basal expression levels of Bcl-2 protein family members, also determined by qRT-PCR (logarithmic scale). Pearson correlation coefficients (r) with associated p values are shown. GPR65 strongly correlates with anti-apoptotic members Bcl-2 (N=87 patients), and also with Mcl-1 and Bcl-xL (N=43 patients). GPR65 does not correlate with pro-apoptotic family members NOXA, PUMA or Bim (N=43 patients).

In each of 87 CLL samples qRT-PCR was used to measure expression levels of GPR65 and various anti-apoptotic and pro-apoptotic Bcl-2 family members. These levels were normalized according to their respective levels in normal B-cells. GPR65 expression levels correlated quite strongly with expression levels of Bcl-2 (r=0.8063, p=<0.001) (Figure 2). GPR65 levels also correlated with levels of two other anti-apoptotic proteins known to play important roles in CLL, Mcl-1 (r=0.4847, p=0.0010) and Bcl-xl (r=0.3411, p=0.0252), although not as strongly as with Bcl-2. GPR65 mRNA expression levels did not correlate significantly with expression levels of BIM, PUMA or NOXA, three pro-apoptotic members of the Bcl-2 family typically expressed in lymphoid malignancies.

### Extracellular acidosis increases Bcl-2 expression in CLL cells

**Figure 3:**
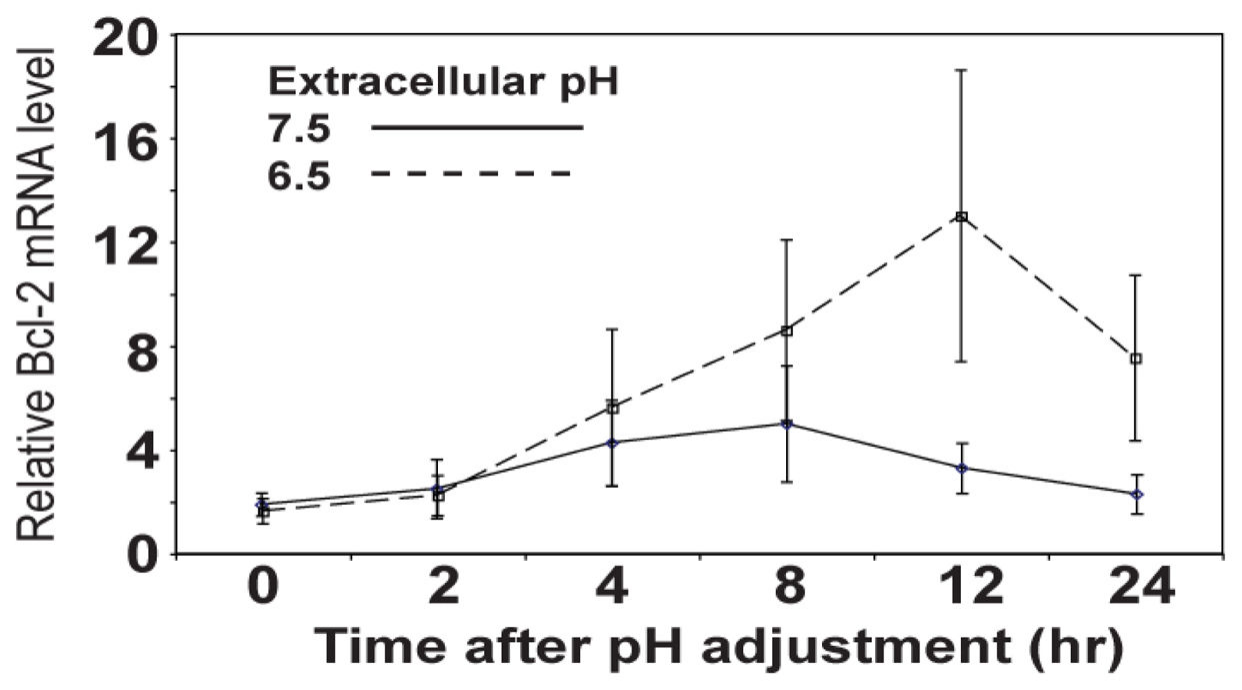
Extracellular acidosis upregulates Bcl-2 expression in primary CLL cells. (A) Bcl-2 mRNA levels were measured by qRT-PCR in three CLL patient samples and cultured at low pH (6.5) and high pH (7.5) for up to 24 hours. Findings indicate that Bcl-2 mRNA levels increase when exposed to extracellular acidosis.

To determine the influence of extracellular acidosis on levels of Bcl-2 and GPR65, CLL cells from 3 patients were cultured under either acidic conditions (pH 6.5) or non-acidic conditions (pH 7.5) for up to 24 hours. Bcl-2 and GPR65 mRNA levels were quantified by qRT-PCR at periodic time intervals. The acidic pH of 6.5 was selected since it approximates the pH at which GPR65 activity is half-maximal [29]. We find that levels of GPR65 in CLL cells are not altered by extracellular acidosis (data not shown), but average levels of Bcl-2 mRNA increase in response to reduced extracellular pH (Figure 3).

**Figure 4:**
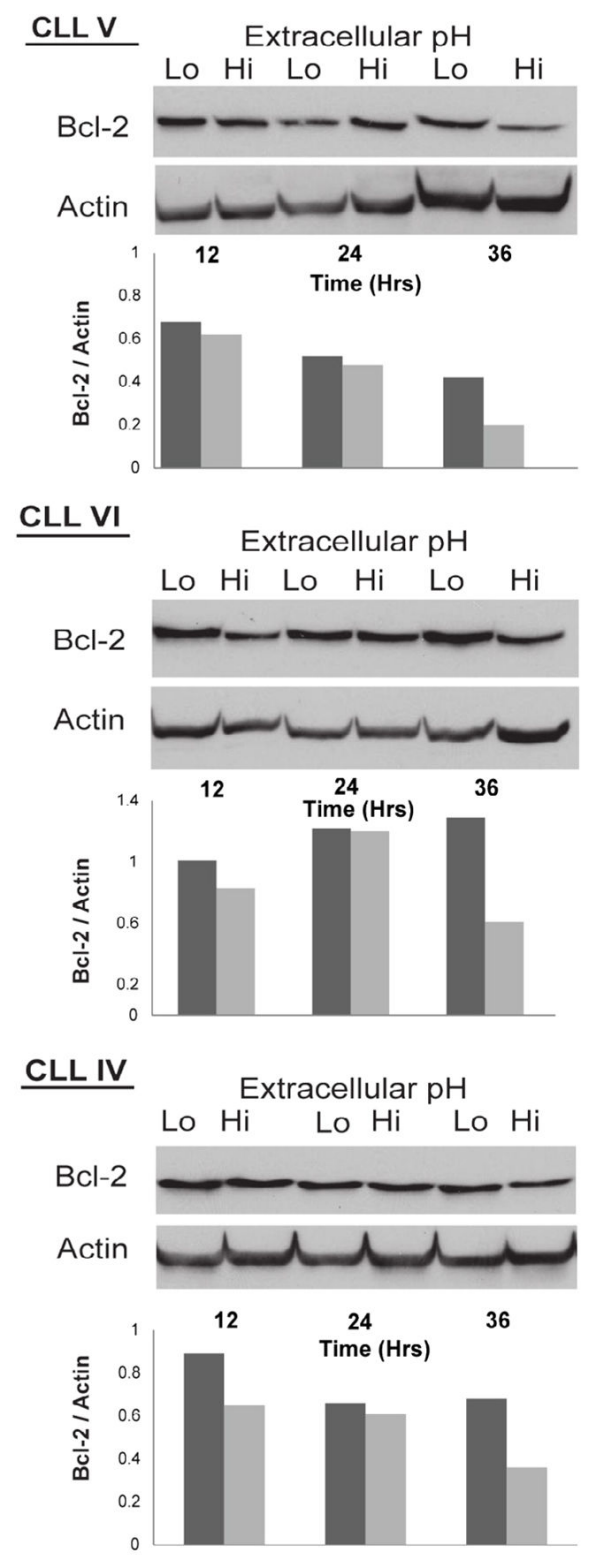
CLL response to extracellular acidosis maintains Bcl-2 expression. Bcl-2 protein levels were measured by immunoblotting in CLL patient samples, arbitrarily labeled by Roman numerals and cultured at low pH (6.5) and high pH (7.5) for the indicated periods of time. Below each panel showing immunoblots is a bar graph in which Bcl-2 protein levels were estimated by densitometry and normalized to actin to control for minor differences in loading at each time point. Findings indicate that the Bcl-2 level in CLL cells declines over time in ex vivo culture, and that this decline is a least partially prevented by extracellular acidosis.

Bcl-2 protein expression was investigated by immunoblotting at baseline (i.e., before pH adjustment) and 12 hour increments in low and control pH. In low extracellular pH, Bcl-2 protein expression was either increased or stable over 36 hours (Figure 4). Under control conditions, Bcl-2 protein expression consistently declined in each patient sample by 36 hours of culture (Figure 4). Thus, from these data we conclude that extracellular acidosis favors up regulation of anti-apoptotic Bcl-2 protein compared to that of control physiologic pH conditions.

## Discussion

The major findings of this study are that the extracellular acidosis sensing receptor, GPR65, is expressed in primary human CLL cells and that its expression levels correlate strongly with expression levels of the anti-apoptotic protein Bcl-2. In addition, we also observe a significant correlation between GPR65 and levels of two other anti-apoptotic proteins, Mcl-1 and Bcl-xl.

These findings raise the possibility that GPR65 signaling may contribute to elevated levels of Bcl-2, at least in a subset of CLL patient samples. We show that GPR65 expression is quite variable (Figure 1) and this may be a reflection of the microenvironment of origin from which the cells were collected: i.e. a more proliferative bone marrow or lymph node vs. a less proliferative bone marrow or lymph node. The mechanism, we postulate, is GPR65-mediated up-regulation of Bcl-2 levels in response to extracellular acidosis in the CLL microenvironment. Although we are unaware of any published, direct measurements of extracellular pH in the CLL microenvironment (i.e., involved lymph nodes or bone marrow), decreased extracellular pH is well documented in proliferative environments associated with the Warburg effect as previously outlined [2,3]. Moreover, in our experiments (Figure 2) extracellular acidosis increases Bcl-2 expression levels in primary CLL cells cultured for brief periods of time ex vivo, suggesting a potential role of acidosis in the microenvironment. In addition, we find that Bcl-2 protein expression levels in three independent CLL samples are maintained following exposure to extracellular acidosis, whereas such levels are attenuated under neutral extracellular pH conditions.

Very little is known about how GPR65 is regulated, including whether the primary mechanisms of regulation are at transcriptional or post-transcriptional levels. Moreover, our findings suggest that GPR65 mRNA levels vary widely among different CLL patient samples. Curiously, we did observe a correlation between GPR65 mRNA levels and the presence of the 13q chromosome deletion, present in more than half of CLL cases. This observation, combined with the observed correlation between GPR65 mRNA levels and Bcl-2 mRNA levels raises interest in the potential role of microRNAs in the regulation of GPR65 expression, particularly in view of previous evidence that miR-15a and miR-16 are frequently deleted or down regulated in CLL samples with 13q14 deletions, contributing to elevated expression of Bcl-2 [30–34]. Utilizing publicly available microRNA databases, we find evidence that GPR65 expression may be up-regulated in response to miR-15a deficiency [35, 36]. On the other hand, another publically available microRNA database predicts GPR65 expression to be targeted by miR-155 [37]. This miR has been shown to be significantly overexpressed by greater than five-fold in patients with CLL [30]. Since miRs are generally negative regulatory elements, this finding might predict an inhibitory effect of miR-155 on GPR65 expression. Thus, the expression level of GPR65 in an individual patient sample may depend upon the relative balance between levels of miR-15a and miR-155. This possibility may explain the broad range of GPR65 levels observed among the individual patient samples included in the present study.

One of the limitations of the present study is the lack of a suitable quality antibody to GPR65. We have extensively tested commercial antibodies available at the time of publication and purported to react with GPR65, but have been unable to verify any of these antibodies. Thus, our measures of GPR65 expression reported here are limited to qRT-PCR estimates of mRNA levels, rather than actual protein levels. Highly sensitive and specific antibodies to GPR65 are needed for future studies to be carried out, with the long-range goal of employing GPR65 as either a novel tumor marker or novel therapeutic target. With regard to these goals, the patient population represented in this preliminary investigation included mainly low-risk, good-prognosis patients, where no significant correlation was detected between relative GPR65 expression levels and known prognostic markers, other than the 13q chromosome deletion. A larger number of patients with more aggressive CLL will likely be needed to determine if GPR65 expression has an impact on the course of this malignancy.

Regardless of these current limitations, the present findings raise the possibility that GPR65 signaling may contribute to the survival potential of CLL cells in the microenvironment. Investigation of the CLL tumor microenvironment has recently come of age. Many studies have also recognized that the poor survival of CLL cells *in vitro* has been hindered by the absence of accessory cells and/or a stromal network to support the survival and proliferation of these cells [38,39]. This observation has led investigators to further examine how the tumor microenvironment influences CLL cell survival. Our investigation focuses on extracellular acidosis as an important moderator of CLL cell survival mediated by the acid sensing receptor GPR65. The extracellular pH has classically been described in solid tumors with values that are often below 7.0 but have been reported with pH values 6.0 or less [12,40]. Although measurements of extracellular pH in the microenvironment of hematologic malignancies are limited, there is now abundant evidence of the importance of the extracellular environment in lymphoid malignancies, particularly CLL [12,41,42].

Future work is needed to characterize the role of GPR65 in more aggressive variants of CLL, where novel prognostic markers and therapeutic approaches are most applicable. It is our hope that this report will stimulate interest in the role of GPR65 in CLL and potentially other lymphoid malignancies. Understanding mechanisms by which CLL cells thrive within the acidic microenvironment may lead to future targeted agents to antagonize GPR65 signaling.

## Figures and Tables

**Table 1:** Clinical and biologic characteristics of CLL patients. Most patients were early Rai stage, with favorable prognostic markers, adequate circulating lymphocytes and being managed with observation alone.

No. patients	76
Sex (female/male)	44/32
Median (range)	
Age (years)	67 (43 – 99)
White blood cells (10^9^/L)	40.8 (3.6 – 400)
Absolute Lymphocyte Count (10^9^/L)	32.30 (0 – 394)
No. (%)	
Rai Stage	
0	27 (36)
1	24 (32)
2	6 (8)
3	5 (6)
4	12 (16)
CD38 Status (60/76 patients)	
CD38 Positive	25 (42)
CD38 Negative	35 (58)
Zap 70 Status (50/76 patients)	
Zap70 Positive	21 (42)
Zap 70 Negative	29 (58)
Trisomy 12 (48/76 patients)	9 (20)
Deletion 13q (49/76 patients)	26 (53)
No Prior Therapy	43 (57)

**Table 2:** Association between clinical characteristics and GPR65 expression. Simple linear regression analysis demonstrates GPR65 expression has correlation with a favorable prognostic marker of 13q deletion. Other prognostic factors show no relationship with GPR65 expression. (ALC: Absolute Lymphocyte Count, SE: Standard Error, CI: Confidence Interval).

	No. Patients	Regression Coefficient	SE	p	95% CI
**Age**	76	0.0080	0.014	0.571	−0.02 – 0.04
**Male**	76	0.1693	0.362	0.641	−0.55 – 0.89
**Stage**	74	−0.1270	0.125	0.311	−0.38 – 0.12
**WBC**	75	0.0009	0.002	0.696	−0.004 – 0.006
**ALC**	75	0.0007	0.002	0.762	−.036 – 0.45
**Zap 70 Positive**	50	−0.6658	0.425	0.124	−1.52 – 0.19
**CD38 Positive**	60	0.4405	0.387	0.259	−0.33 – 1.21
**Trisomy 12**	48	−0.3971	0.588	0.503	−1.58 – 0.77
**Deletion 13q**	49	0.9739	0.431	0.029	0.11 – 1.84
